# Size does not matter: commensal microorganisms forge tumor-promoting inflammation and anti-tumor immunity

**DOI:** 10.18632/oncoscience.145

**Published:** 2015-03-23

**Authors:** Melanie R. Rutkowski, Jose R. Conejo-Garcia

**Affiliations:** ^1^ Tumor Microenvironment and Metastasis Program, The Wistar Institute, Philadelphia, USA

**Keywords:** microbiota, inflammation, cancer, TLR5, gamma delta T cell

## Abstract

Recent studies have demonstrated that the commensal microbiota are indispensable for the maintenance of immune homeostasis, orchestration of immune responses against pathogens and most recently during cancer immunotherapy and malignant progression of extraintestinal tumors. Here we discuss the recent findings that a common genetic variation in TLR5 influences the progression and outcome of ovarian, sarcoma, and luminal breast tumors and the implications of these findings in light of recent publications describing the role of the commensal microbiota in control of the systemic immune system.

## COMMENSAL MICROORGANISMS ARE REQUIRED TO FORGE A ROBUST IMMUNE SYSTEM

Barrier surfaces in humans are colonized with trillions of microorganisms including hundreds of different bacterial and fungal species, as well as viruses and protozoans [1, 2]. These commensal microorganisms colonize the upper respiratory tract, genitourinary tract, skin, and intestines shortly after birth. Only in the intestine, it is estimated that the number of bacteria is around 10**^14^**, outnumbering eukaryotic cells at a ratio of 10:1 [3].

Recent studies have demonstrated that commensal microorganisms are required to maintain the metabolic needs of the host, but also for the immune homeostasis of mucosal surfaces [4, 5]. As importantly, the microbiota also plays a crucial role in the postnatal development of the immune system [6]. Commensal microorganisms contribute to forge the development of a robust immune system that is able to defend the host from infections and tumors at places that are distal from colonized locations [7]. Although our understanding of these mechanisms is in its infancy, the relevance of the microbiota during immune development is evidenced by significant decreases in both conventional CD4 T cells [8] and regulatory T cells [9] in the spleens of germ-free mice, compared to their normally colonized counterparts. In the absence of commensal microorganisms, mice also develop a narrower repertoire of TCR clones [10], and their T cell compartment is severely defective and skewed towards a Th2 response [11]. Correspondingly, microbial colonization of germ-free animals drives an increase in immunoglobulin levels and enhances systemic immune responses [6].

Given the importance of the microbiome in postnatal immune development, the presence ***vs.*** the absence and/or the composition of the microbiota have been found to play a role in different diseases associated with adaptive immune responses. Microbiota-induced IL-1β production, for instance, has been also shown to be required for Th17 differentiation in the intestine [12]. Correspondingly, the course of various models of autoimmunity that rely on IL-17-driven inflammatory responses - including diabetes [13], collagen-induced arthritis [14], or experimental colitis [15] - is significantly influenced by commensal bacteria.

Commensal-derived signals are also required for optimal immune defense against systemic pathogens. Nod1-mediated recognition of commensal-derived peptidoglycan primes neutrophils to rapidly respond to pathogenic bacterial infection [16], whereas during viral infection, the commensal microbiota are required to maintain a basal level of type I IFN signaling that determines the activation threshold of innate immune responses [17]. Correspondingly, a previously unappreciated role in driving protective antibody responses in response to influenza vaccines has also been recently identified for commensal bacteria, whereby, vaccination responses were significantly impaired in germ-free or antibiotic-treated mice and restored by reconstituting the microbiota with flagellated bacteria [18].

## COMMENSAL BACTERIA INFLUENCE SPONTANEOUS TUMOR PROGRESSION AND THERAPEUTIC RESPONSES

A role for the microbiota has also been associated with carcinogenesis through both metabolic alterations and the promotion of inflammatory responses [3]. More importantly, because solid tumors are invariably associated with inflammation, it is not surprising that certain commensal bacterium promote carcinogenesis locally in the colon, where they are more abundant [19]. This is consistent with the role of chronic inflammation in the intestinal tract (e.g., ulcerative colitis) in the promotion of colon cancer, an association known for years. However, until very recently, the role of the commensal microbiota in influencing the progression of systemic extraintestinal cancers has remained unknown. These studies have either focused on the role of immunological defects that result in significant dysbiosis and bacterial translocation to the liver, or have focused on the role of commensal microbiota in the presence of an unharmed mucosal barrier. For instance, emerging evidence suggests that a subset of intestinal NKp46(+) lymphoid tissue-inducer (LTi) cells could play a role in the suppression of (distal) B16 melanoma [20]. Even more recently, Goldszmid, Trinchieri and Zitvogel simultaneously reported for the first time how interactions between microbiota and mucosal surfaces are required for therapeutic responses to different anticancer interventions against tumors occurring outside of the intestinal tract [21-24]. In these seminal reports, it was conclusively demonstrated that chemotherapeutic agents such as oxiplatinum and immunomodulatory agents such as CpGs both require commensal bacteria for inducing tumor rejection, because depletion of commensal bacteria with antibiotics resulted in consistently accelerated malignant progression. However, differential effectiveness is driven by dissimilar mechanisms: myeloid cell-derived TNF-α, produced in response to TLR activation, likely with the contribution of type I IFN, are needed for the curative effects of CpGs combined with anti-IL-10R antibodies, and are dependent on the presence of gut commensal bacteria [23]. In contrast, the optimal anticancer effects of chemotherapeutics such as cyclophosphamide require anti-commensal Th17 and memory Th1 responses, which are facilitated by enhanced intestinal permeability and subsequent bacterial translocation caused by these drugs [22, 23].

The obvious implication of these studies is that antibiotics can compromise the efficacy of certain anti-cancer treatments. However, in the absence of treatment, we have demonstrated that the progression of tumor models of sarcoma, cervical cancer and ovarian carcinoma is significantly delayed when intestinal bacteria are depleted with a cocktail of broad-spectrum antibiotics [25]. Our study shows that the microbiota, in a TLR5 signaling-dependent manner, drives the up-regulation of tumor-promoting IL-6 in the serum of tumor-bearing mice, subsequently promoting MDSC mobilization [25, 26]. Expanded MDSCs in turn influence γδ T cells to secrete immunosuppressive galectin-1, which abrogates anti-tumor immunity and accelerates malignant progression.

In contrast, there is a second class of tumors characterized by relatively low production of IL-6 and exemplified by luminal (but likely not basal) breast cancer. In these tumor systems, depletion of commensal bacteria in wild-type mice accelerates extra-mucosal tumor growth [25]. Interestingly, blockade of tumor-promoting IL-17 in the absence of high levels of IL-6 significantly delays malignant progression, while the same intervention has no effect when serum IL-6 levels are higher [25].

Overall, our results underscore the critical role of commensal bacteria in tumor progression, by governing the orchestration of tumor-promoting inflammation. Inflammatory responses depend on both hematopoietic cells and the capacity of tumor cells to secrete IL-6 in an autocrine and paracrine manner. Correspondingly, preventing the capacity of tumor cells to up-regulate IL-6 through different shRNA clones is sufficient to: 1) significantly decrease serum IL-6 levels; 2) diminish MDSC mobilization; 3) reverse the galectin-1-dependent regulatory activity of γδ T cells; and 4) render previously IL-17 insensitive tumors susceptible to IL-17 blockade. Most importantly, although bigger cohorts of patients and independent datasets are needed to indisputably confirm the relevance of these findings, our data suggest that (IL-6^hgih^) ovarian and (IL-6^low^) luminal breast cancer patients recapitulate the mechanisms identified in our mouse models [25].

## SPECIFIC DEFFECTS IN INNATE IMMUNITY PROMOTE BACTERIAL TRANSLOCATION AND DYSBIOSIS

An important consideration to understand the role of the microbiota in health and disease is how differences in the composition of the commensal microflora could influence experimental differences across different laboratories. The occurrence of colitis in the absence of certain immunological sensors in some facilities, for instance, has been also reported as a major contributor to accelerated malignant progression. However, the mechanisms driving accelerated tumor growth under these conditions are very different from the ones that we observed in tumor-bearing hosts with a preserved mucosal barrier. For instance, defects in various components of the inflammasome, including Nlrp6, Nlrp3 and Capase-1 cause dysbiosis and intestinal inflammation in some facilities [27-29]. This results in microbial products leaking through the portal vein to the liver, where they activate inflammation and promote fatty liver disease, with dramatic metabolic changes in the host.

Some important factors that impact microbiome-dependent mechanisms in multiple ways include the hygiene of the facility; the nature of the diet; and the pH of drinking water. The incidence and progression of diabetes in NOD mice, for instance, are profoundly influenced by the administration of acidic ***vs.*** neutral water [30, 31]. Mice receiving acidic water mice also experienced significant changes in their bacterial repertoire, including an increase in the abundance of Bacteroides. This may contribute to explain experimental differences in the occurrence of a metabolic syndrome across different vivariums, because certain laboratories (e.g., the Jackson Laboratory) provide their mice with acidified drinking water, safeguarding the cleanliness of the water and reproducibility across experiments with the consequence of altering the microbiome. In contrast, mice receiving neutral water could have alterations to their microbiota, depending on the hygiene of the facility and contaminants in their drinking bottles. For instance, we [25] and others [32] have not observed any evidence of intestinal inflammation, metabolic syndrome or measurable bacterial translocation across the mucosal barrier in tumor-bearing mice housed in our facilities, including TLR5-deficient animals. In fact, the signal of PCR amplification of 16S ribosomal RNA at tumor, lymphatic or other non-mucosal locations was never higher than the signal detected in wild-type tumor-free mice [25]. Although we cannot rule out that bacterial products still translocate and have a significant effect in the activation of distal immune cells, we were unable to detect measurable levels of endotoxin in serum or tumor ascites. In contrast, an obvious metabolic syndrome and the presence of colitis and significant increases in the levels of IL-1β within their colons was confirmed in the same TLR5**^−/−^** mouse housed in different facilities [33-35].

## GENETIC VARIATION IMPACTS THE RESPONSE TO COMMENSAL MICROORGANISMS

Interactions between the microbiome and mucosal surfaces are regulated in part by stimulation of innate immune “pattern recognition receptors” (PRRs). PRRs are proteins that recognize pathogen-associated molecular patterns (PAMPs), associated with microorganisms or damage-associated molecular patterns (DAMPS), associated with cellular stress. In mammals, these molecules can be broadly classified into membrane-bound PRRs, which include Toll-like receptors (TLRs) and the mannose receptor; and cytoplasmic PRRs, which include NOD receptors, NALPs and RNA Helicases.

Interestingly, At least 30% of individuals in the general population are carriers of polymorphisms in PRR genes. For instance, up to 23% of individuals in the general population have detrimental mutations affecting cell-surface TLRs [36]. In addition, 16% of individuals carry nonsense mutations in at least one of these TLRs. The most frequent loss-of-function for any TLR is associated with the R392X variant of TLR5, which has a dominant-negative effect and affects ~7-10% of the general population [37]. TLR5 is the pathogen recognition receptor (PRR) for flagellin, which is exclusively a bacterial product. Functional TLR10 polymorphisms are also very frequent, affecting up to 5% of the general population. Another very frequently altered PRR is NOD2/CARD15. Polymorphisms in the 3 most common hotspots are as frequent as 7.8% in Caucasians [38], and confer a risk for the development of Crohn's disease.

Multiple polymorphisms of functional relevance are also found in other important viral sensors such as the RNA helicase RIG-I. The most significant effect in function occurs due to a SNP in codon 183 (S>I), which induces an abortive conformation of RIG-I, rendering it incapable of downstream signaling [39]. However, the frequency of this polymorphism in the general population is unknown.

These frequent deficiencies in PRRs are obviously compatible with a healthy life, which indicates lack of evolutionary pressure and suggests a redundant biological role. However, some of these polymorphisms have immunological consequences, because they alter the susceptibility of the host to develop certain diseases. For instance, TLR5-deficient individuals are more susceptible to develop pneumonia caused by Legionella pneumophila [37], and are less likely to develop lupus. In contrast, 32% of patients with Chron's disease have common NOD2 polymorphisms, which is associated with more aggressive disease [40].

Our recent studies indicate that the dominant negative R392X TLR5 polymorphism determines the nature of tumor-promoting inflammation in the presence of a tumor at sterile (extra-mucosal) locations [25]. In tumor-bearing individuals, through interactions between the microbiome and TLR5^+^ cells that occur at places of bacterial colonization, TLR5 competence drives systemic IL-6 up-regulation, while deficiencies in TLR5 signaling are associated with higher circulating levels of IL-17. We know that flagellated bacteria and hematopoietic TLR5^+^ cells at mucosal surfaces are driving differential tumor progression because: 1) depletion of commensal bacteria with a non-absorbable cocktail of antibiotics abrogates any TLR5-dependent differences in tumor growth; 2) and reconstitution of TLR5^+^ mice with TLR5-deficient (but not TLR5^+^) bone marrow recapitulated the delayed progression of syngeneic and autochthonous tumors observed in TLR5 KOs. Paradoxically, we have shown that acute stimulation of TLR5 with flagellin boosts anti-tumor immunity in established ovarian cancer models [41].

Overall, our data illustrate the previously unrecognized importance of genetic variation in PRRs in the orchestration of tumor-promoting inflammation through interactions between the microbiome and the immune system, and open new avenues to understand dissimilar responses as a function of genetic factors.

## THE REPERTOIRE OF COMMENSAL BACTERIA DETERMINES INFLAMMATORY RESPONSES AND IS INFLUENCED BY GENETIC FACTORS

Surprisingly, our studies have also identified that at least TLR5 deficiency drives significant and intrinsic differences in the composition of the microbiome [25]. Thus, these differences did not merely reflect divergence of the microbiota after long-term husbandry in isolation from each other, as previously reported [42]. Most shockingly, when TLR5-competent and TLR5-deficient mice were co-housed together for >4 weeks, variances in the composition of the microbiota remained, and differential malignant progression continued [25]. Because mice are coprohagic, co-housing should homogenize their microbiome if its composition is merely dependent on environmental conditions. However, our results illustrate that PRR-dependent changes in the host response are sufficient to alter the repertoire of at least commensal bacteria, independently of the effects of long-term co-housing of wild-type and mutant mouse strains [42]. Thus, we observed significant increases in the abundance of actinobacteria and firmicutes in TLR5-deficient mice, while bacteroidetes were expanded in TLR5-competent co-housed littermates [25]. Nevertheless, we found much more dramatic compositional differences in wild-type mice housed in a different vivarium, further illustrating how facility-dependent differences of animal husbandry may explain experimental differences in multiple studies as a result of dissimilar microbiomes, and urging caution in the interpretation of data that could be influenced by the microbiome.

The repertoire of mucosal microorganisms is important because certain strains favor particular inflammatory responses. For instance, seminal studies by the Littman laboratory demonstrated that segmented filamentous bacteria (SFB) drive the expansion of IL-17 producing cells [43]. Interestingly, we did not observe significance differences in SFB between TLR5-deficient and wild-type mice, despite the fact that they exhibit significantly higher serum levels of IL-17, at least in the presence of a tumor [25]. In contrast, other bacterial strains, including Bacteroides fragilis [4] and some Clostridia [44] promote the generation of regulatory T cells that dampen inflammation through the secretion of IL-10.

Because most bacteria colonizing with the intestines of healthy humans are included in one of four main phyla (Firmicutes, Bacteroidetes, Actinobacteria and Proteobacteria) [6], understanding the different contribution of each strain to the orchestration of systemic inflammatory responses and immune homeostasis could open new therapeutic avenues. Thus, adjusting the composition of the microbiome through antibiotics and oral enrichment of certain bacterial strains is a reality for the treatment of inflammatory diseases and could contribute to modulate inflammatory responses also in cancer patients. The feasibility of targeted microbiome reconstitution has recently been demonstrated to treat individuals that are susceptible for infection with ***Clostridium difficile*** [45].

Furthermore, while most studies have so far focused on the role of gut bacteria, much less is known about how commensal fungi or viruses influence biological processes, including malignant progression. Future studies should help to clarify the role of these additional components of the microbiome.

## DOUBLE-EDGE γδ T CELLS INFLUENCE MALIGNANT PROGRESSION IN A MICROBIOTA-DEPENDENT MANNER

Another unexpected novelty of our recent studies is a previously unrecognized contribution of γδ T cells to the immunosuppressive burden in the microenvironment of different tumors, which is dependent upon the interactions of TLR5^+^ immune cells with the microbiota. γδ T cells are relatively abundant in solid ovarian or breast cancers of human origin, typically representing >6% of total leukocytes and outnumbering Foxp3^+^ regulatory T cells [25].

Although the immunosuppressive activity of γδ T cells infiltrating breast cancer specimens was previously reported, those studies involved the use of selected clones [46]. In addition, the mechanisms of effector T cell suppression remained elusive. Our work demonstrates that bulk populations of γδ T cells in human and mouse tumors secrete more immunosuppressive galectin-1 on a per cell basis than any other cell type in the tumor microenvironment. Correspondingly, galectin-1 selectively secreted by tumor-derived γδ T cells was sufficient to promote accelerated tumor growth [25].

Galectin-1 is emerging as a major accomplice in malignant progression, through a variety of mechanisms that involve not only immunosuppression [47], but also angiogenesis [48] and the promotion of metastasis [49]. Relevant for its regulatory activity, secreted galectin-1 cross-links cell-surface glycoconjugates bearing multiple units of the N-acetyllactosamine (Galβ1-4-NAcGlc) disaccharide and abrogates potentially protective Th1 and CD8 effector T cell responses [50, 51]. Galectin-1 is highly abundant in ovarian cancer and there are multiple cellular sources, but our studies identify γδ T cells as major contributors to the immunosuppressive burden through the production of this crucial mediator.

**Figure 1:**
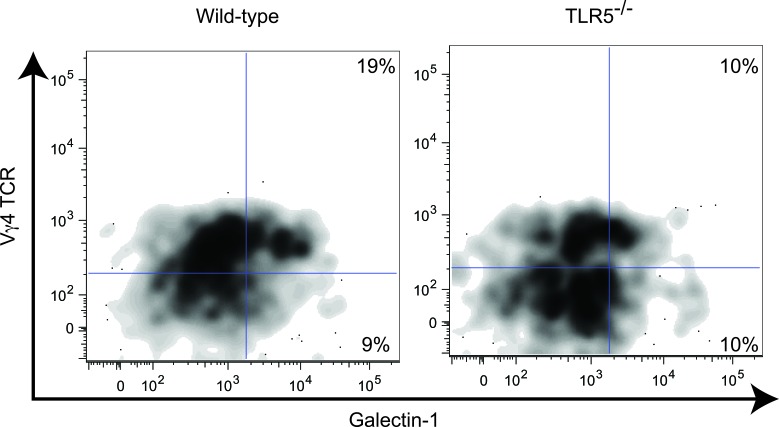
Galectin-1 producing γδ T cells are predominantly Vγ4 TCR+ Intracellular cytokine staining of γδ T cells from the tumor-draining lymph nodes of wild-type or TLR5^−/−^ tumor-bearing mice. Plots are gated on Live, CD45^+^, CD3^+^, γδ TCR^+^. Numbers represent the proportion of γδ T cells that are galectin-1^+^ and Vγ4 TCR^+^.

γδ T cells are significantly more abundant at mucosal locations and therefore the influence of the microbiota in their activities was not unexpected. However, perhaps one of the most intriguing aspects of our work is that the acquisition of regulatory attributes by γδ T cells appears to take place at extra-mucosal locations and not locally at places of direct interactions with the microbiota. This is supported by the highest producers of galectin-1 in the tumor microenvironment in γδ T cells carrying the Vγ4 (Figure 1) and, to a lesser extent, the Vγ2 (not shown) chains in mice, thus corresponding to subsets primarily located in the periphery rather than in the intestine. In addition, γδ T cells sorted from the lymph nodes of healthy mice can be induced to secrete immunosuppressive galectin-1 in response to adenosine, which is generated at high levels by granulocytic (but not myelomonocytic) MDSCs [25]. Consequently, the suppressive activity of γδT cells is entirely dependent upon TLR5 signaling by commensal bacteria, so that γδ T cells in TLR5-deficient tumor-bearing mice paradoxically show protective activity [25].

Future studies will help to establish the plasticity of γδ T cells and the environmental cues required for the induction of immunostimulatory or immunosuppressive functions. Furthermore, it would be beneficial to define the full spectrum of functional activities of γδ T cells in ovarian cancer to enhance the potential of targeting or transforming them through novel interventions. For example, because our lab has recently demonstrated that FoxP1 is fundamental for TGFβ immune-mediated suppression in activated T cells [51], it would be interesting to determine whether targeting FoxP1 in ex vivo expanded γ T cells would enhance their anti-tumor effector functions.

## CONCLUSIONS AND FUTURE DIRECTIONS

Humans are symbiotic organisms colonized by trillions of microbes. In recent years, it has become clear that the composition and abundance of the microbiome determines multiple physiological and pathological processes, including autoimmunity, responses to vaccination and infections, and cancer progression and therapy. A better understanding of the role of individual strains of bacteria during various inflammatory conditions could provide a means of mediating or abrogating systemic inflammation through the modulation of the composition (or the total burden) of commensal microorganisms. This will need to take into account the importance of genetic variation in interactions between the microbiome and mucosal surfaces, and in particular the role of frequent functional polymorphisms in PRRs. A better understanding of how the microbiota influences therapeutic responses ***vs.*** spontaneous malignant progression in different types of cancer will also provide a rationale for antibiotic treatment and supplementation of more protective bacterial strains.

Many other open questions should be addressed in the next years. Among them, the role of commensal microorganisms different from bacteria (e.g., fungi or viruses) in inflammatory and adaptive immune responses, including those triggered by tumors. Furthermore, while it has been recently shown that the major human commensal bacterial phyla are resistant to the effects of inflammation-induced antimicrobial peptides [52], much more remains to be understood about whether other biological processes that alter the immune system (including cancer) influence the composition of the microbiota and, subsequently, other physiological and pathological events. Finally, why some inflammatory cytokines are produced in the presence of a tumor and are dependent upon the commensal microbiota needs to be further addressed.

Upcoming developments will clarify these and other issues and will provide a mechanistic rationale for the design of novel anti-cancer interventions.
